# Pressure-controlled ventilation versus volume-controlled ventilation for adult patients with acute respiratory failure: A systematic review and meta-analysis

**DOI:** 10.1097/MD.0000000000043774

**Published:** 2025-08-22

**Authors:** Sitta Suntrawanichakul, Chetta Ngamjarus, Kittisak Sawanyawisuth, Sittichai Khamsai

**Affiliations:** a Faculty of Medicine, Khon Kaen University, Khon Kaen, Thailand; b Department of Epidemiology and Biostatistics, Faculty of Public Health, Khon Kaen University, Khon Kaen, Thailand; c Department of Medicine, Faculty of Medicine, Khon Kaen University, Khon Kaen, Thailand.

**Keywords:** barotrauma, controlled ventilation, pneumothorax, respiratory failure, systematic review

## Abstract

**Background:**

There is limited data on the effectiveness of pressure-controlled ventilation (PCV) and volume-controlled ventilation (VCV) in adult patients with acute respiratory failure. This study aimed to compare the effectiveness of these 2 ventilations.

**Methods:**

We performed a comprehensive search of 4 electronic databases, including PubMed, Central, Scopus, and CINAHL, from inception to July 14, 2023. This systematic review included randomized controlled trials that compared VCV and PCV ventilator modes in intubated adult patients with acute respiratory failure from any condition. Eligible studies were evaluated for study characteristics and outcomes. Details of study characteristics included authors, publication year, country, study aims, study design, study population, and characteristics of eligible patients: age, sex, disease severity, and comorbidities. The outcomes of interest were the incidence of barotrauma or pneumothorax and the in-hospital mortality rate. Meta-analysis with a fixed-effect model was used to pool the results of included studies.

**Results:**

There were 27 articles that were eligible; 4 articles met the study criteria. These studies included acute respiratory distress syndrome patients (3 studies) and chronic obstructive pulmonary disease patients with open heart surgery. The total patients in the VCV and PCV groups were 581 and 548, respectively. There was no significant difference in the incidence of barotrauma between the VCV and PCV groups (risk ratio = 0.79, 95% confidence interval: 0.56–1.12). The VCV group had a slightly higher mortality rate than the PCV group (risk ratio = 1.15, 95% confidence interval: 1.00–1.33).

**Conclusions:**

PCV and VCV had no significant difference in both barotrauma incidence and mortality rate. PCV mode may have slightly lower mortality and may be a preferable ventilator mode in patients with acute respiratory distress syndrome. Further included studies may be required to confirm the results of this study.

## 1. Introduction

Acute respiratory failure is a serious medical condition associated with high morbidity and mortality. A previous U.S. study reported that its incidence increased from 245 cases per 100,000 adults in 2002 to 455 cases per 100,000 adults in 2017.^[1]^ Although mortality rates declined from 34% to 23% over this 15-year period, the average hospital charge remained high at $158,433 USD. Despite this improvement, survivors often face long-term consequences, including mental, physical, social, and functional impairments such as anxiety and depression.^[2]^

Mechanical ventilation is a lifesaving intervention for patients with acute respiratory failure.^[1]^ Mortality rates among those receiving invasive mechanical ventilation decreased from 37% in 2002 to 30% in 2017, regardless of the underlying cause. The common ventilation modes include pressure-controlled ventilation (PCV) and volume-controlled ventilation (VCV).^[3]^ These modes differ in their advantages and risks, including the incidence of barotrauma, pneumothorax, and mortality. A 2015 systematic review comparing PCV and VCV, which included only 3 randomized controlled trials (RCTs), found no conclusive evidence favoring either mode.^[4]^ The risk ratio (RR) for hospital mortality was 0.83 (95% CI = 0.67–1.02), but the analysis was limited to patients with acute lung injury or acute respiratory distress syndrome (ARDS).^[4]^ Adding more respiratory settings may broaden usage of study results. This study aimed to compare the effectiveness of the 2 ventilations (PCV vs VCV) in adult patients with any causes of respiratory failure in terms of incidence of barotrauma, pneumothorax and in-hospital mortality rate.

## 2. Methods

### 2.1. Inclusion criteria

This systematic review included any RCTs compared VCV versus PCV ventilator mode in intubated adult patients with acute respiratory failure from any conditions. The other study designs were excluded including observational studies, case reports, case series, or review articles. Additionally, studies conducted in 1 lung ventilation, no comparison between VCV versus PCV, using PCV/VCV in the same patients, no outcomes reported, or non-English studies were excluded.

### 2.2. Search methods and studies selection

CN did a comprehensive search of PubMed, Central, Scopus, and CINAHL from inception to July 14, 2023. Search terms included artificial respiration, mechanical ventilation, volume control, VCV, pressure control, PCV, pneumothorax, and barotrauma. The details of search strategies were in the Appendix, Supplemental Digital Content, https://links.lww.com/MD/P614. All records for search results from above databases were combined and remove any duplicated records using Mendeley software.^[5]^ Then, title and abstract of each article was screened by SS and SK independently. SS and SK independently read full texts for identifying included studies.

### 2.3. Data extraction and outcomes

Eligible studies were evaluated for study characteristics and outcomes. Details of study characteristics included authors, publication year, country, study aims, study design, study population, and characteristics of eligible patients: age, sex, disease severity, and comorbidities. The outcomes of interest were the incidence of barotrauma or pneumothorax and in-hospital mortality rate.

### 2.4. Risk of bias assessment

We evaluated the risk of bias assessment of included studies follow the Cochrane handbook of systematic review of interventions that we had to assess 6 domains including random sequence generation, allocation concealment, blinding of participants and personnel, blinding of outcome assessment, incomplete outcome, selective reporting, and other bias.^[6]^ All domains were evaluated and reported as low risk, unclear or high risk. The assessment was independently performed by SS and SK.

### 2.5. Statistical analysis

Data from included studies were extracted and inputted in RevMan 5.^[7]^ RR with its 95% confidence interval (CI) of interesting outcomes were calculated and presented in the forest plots. We used the Fixed-effect model to pool results with the same outcome from included studies because there was no substantial heterogeneity between studies results (*P*-value of Cochran *Q* statistic >.1 and *I*^2^ statistic < 50%). The characteristics of included studies were summarized and reported. The incidence of barotrauma or pneumothorax was reported in number and percentage in the VCV and PCV groups as well as the in-hospital mortality rate.

### 2.6. Ethical consideration

Ethical approval was waived as this study did not involve humans or animals.

## 3. Results

**Figure 1.**
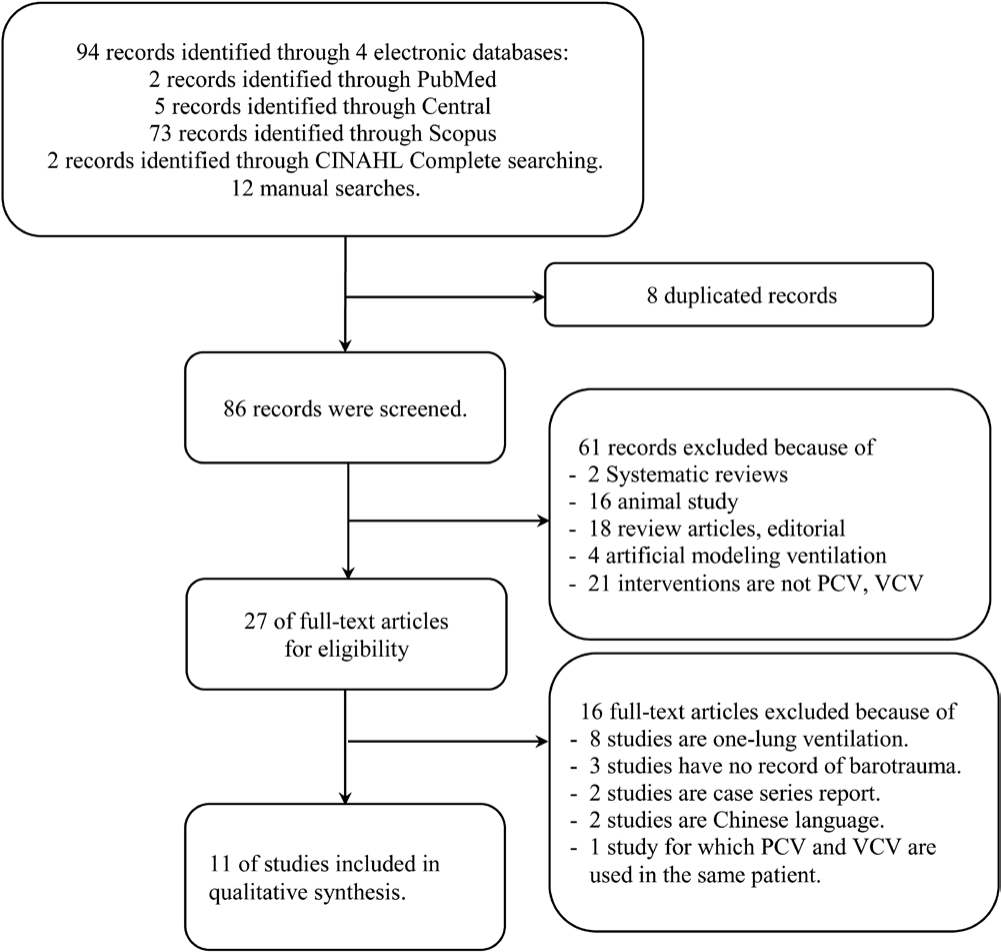
A prisma flow diagram.

There were 94 records identified from search results (Fig. 1). Of those, 86 records were screened after an exclusion of duplication (8 records). There were 27 articles were eligible for full text review and 4 studies were met our eligibility criteria and included this review.^[8–11]^ Short reasons of exclusion of 23 articles were shown in Figure 1; mostly due to 1 lung ventilation or not a comparison study between PCV and VCV.

The 4 included studies were published between 1994 and 2014 and conducted in the US, Spain, Türkiye, and 1 international study in Canada, Australia, and Saudi Arabia (Table 1). Three studies collected data from ARDS patients^[8–10]^ and 1 study included COPD patients with open-heart surgery.^[11]^ The total patients in the VCV and PCV groups were 581 and 548 patients, respectively or total of 1129 patients. The mean age of patient in these studies was ranging from 43.1 to 59 years with a slightly higher proportion of male patients (686 patients; 60.76%).

**Table 1:** Study characteristics of included study compared PCV and VCV in patients with acute respiratory failure.

Study	Year	Country	Study aim	Study design	Population (n)	Male: female	Age, mean (SD)	Initial severity stratification, mean (SD)	Comorbidities
Esteban	2001	Spain	To compare in-hospital mortality of patients with ARDS ventilated with PCV or VCV with a square-wave inspiratory flow	RCT, 12 ICU (Multicenter)	ARDS patient VCV (n = 42) PCV (n = 37)	30: 12 24: 13	59 (16) 56 (17)	SAPS II score 42 (17) 39 (14)	Cardiovascular failure, renal failure, liver failure, coagulation failure, digestive failure, metabolic failure, CNS failure, sepsis, shock, pneumonia, multiple transfusions, pulmonary contusion, multiples fractures, gastric aspiration, acute pancreatitis, near-drowning
Maede	2008	Canada, Australia, Saudi Arabia	To compare an established low-tidal-volume ventilation strategy with an open-lung approach strategy; combining low-tidal volume, lung recruitment maneuvers, and high positive-end–expiratory pressure	RCT, 30 ICU (Multicenter)	ARDS patient VCV (n = 508) PCV (n = 475)	307: 201 282: 193	56.9 54.5	APACHE II score 25.9 24.8	Sepsis, pneumonia (non-PJP), gastric aspiration, multiple transfusion, Prolonged shock, pulmonary contusion, multiple major fractures, acute pancreatitis, drug overdose, PJP, burn injury, inhalation injury
Rappaport	1994	US	To assess the feasibility and utility of early and sustained use of pressure-limited ventilation, compared with volume-limited ventilation, in patients with severe respiratory failure	RCT	ARDS patient VCV (n = 11) PCV (n = 16)	7: 4 9: 7	51.6 (6.3) 43.1 (4.3)	APACHE II score 21.3 (1.58) 21.3 (1.44)	Sepsis, pneumonia, drug reaction, CNS injury
Ugurlucan	2014	Turkey	To compare mortality and morbidity risk of using PCV and VCV in the postoperative period of open-heart surgery for COPD patient.	RCT	COPD patient who undergoing open-heart surgery VCV (n = 20) PCV (n = 20)	13: 7 14: 6	52.7 (8.4) 54.7 (11.3)	FEV_1_, % 44.7 (4.9) 45.2 (5.1)	Hypertension, smoking history, diabetes mellitus, carotid or peripheral arterial disease, remote MI (>1 mo)

APACHE = acute physiology and chronic health evaluation, ARDS = acute respiratory distress syndrome, CNS = central nervous system, COPD = chronic obstructive airway disease, FEV1 = force expiratory volume in 1 s, MI = myocardial infarction, PJP = pneumocystis jiroveci pneumonia, RCT = randomized controlled trial, SAPS = simplified acute physiology score.

**Figure 2.**
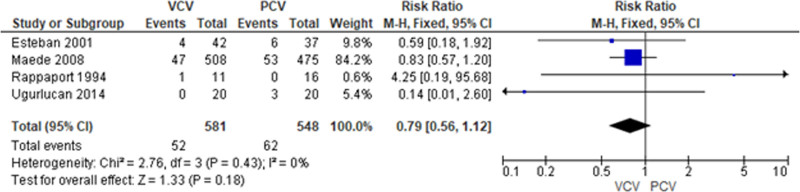
Barotrauma incidence between PCV versus VCV in patients with acute respiratory failure. PCV = pressure-controlled ventilation, VCV = volume-controlled ventilation.

**Figure 3.**
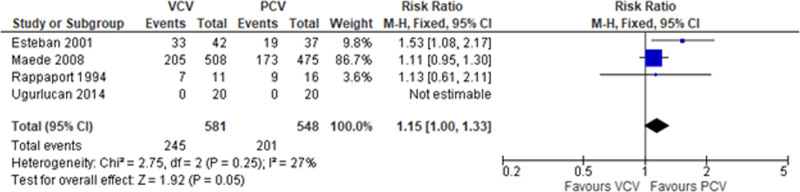
Mortality rate between PCV versus VCV in patients with acute respiratory failure. PCV = pressure-controlled ventilation, VCV = volume-controlled ventilation.

Regarding outcomes, the incidence rate of barotrauma in the VCV and PCV group was 8.95% (52 out of 581 patients) and 11.31% (62 out of 548 patients), respectively. The VCV group had lower incidence of barotrauma when compared with the PCV group with RR 0.79 (4 studies, 95% CI: 0.56–1.12) as shown in Figure 2. The VCV group had slightly higher mortality rate than the PCV group (42.17% vs 36.68%); RR = 1.15 95% CI: 1.00–1.33 as shown in Figure 3.

**Figure 4.**
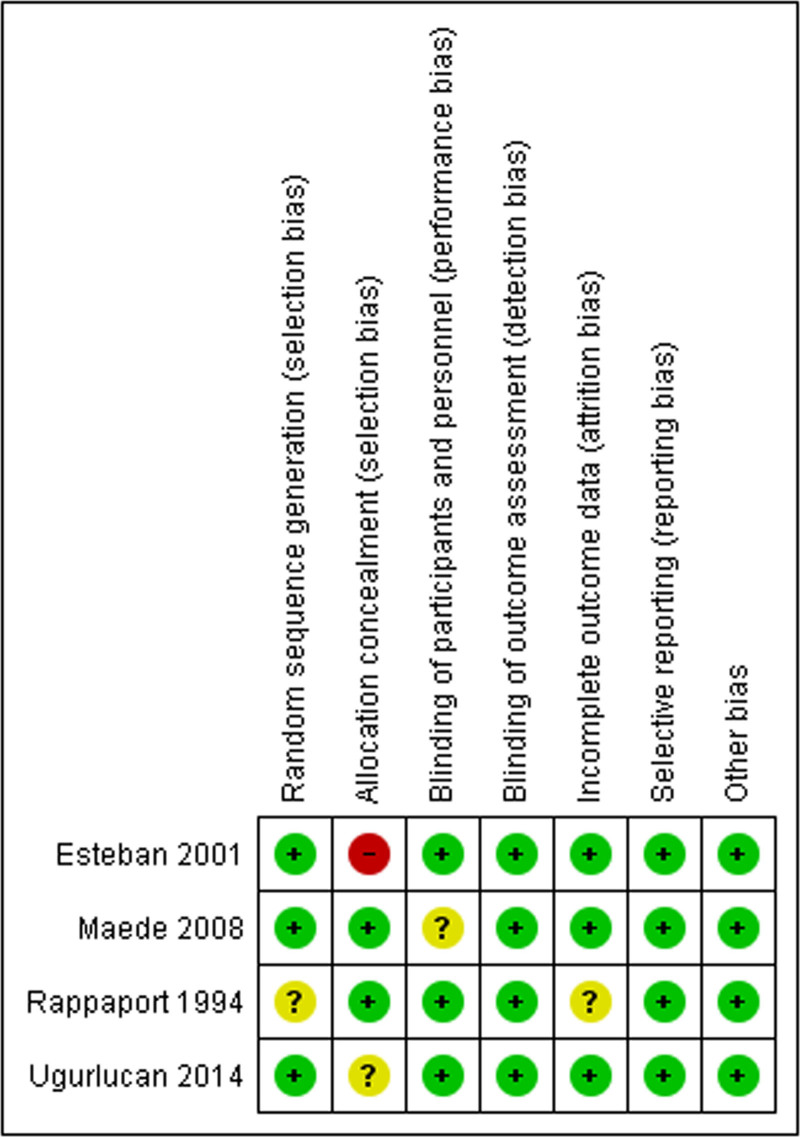
Risk of bias assessment of included studies.

The 4 included showed low risk for publication biases assessed by using the bias assessment criteria for randomized controlled trial (Fig. 4). Only the Esteban study had high risk of bias in the allocation concealment or selection bias (Fig. 4).

## 4. Discussion

There are several ventilator induced lung injury from invasive mechanical ventilators including barotrauma, lung deflation injury, or effect-induced lung injury.^[12]^ A previous published study found that using low-tidal volume of 6 mL per kg of predicted body weight with a plateau pressure of 30 cm of water or less had significantly lower mortality rate than the tidal volume of 12 mL per kg of predicted body weight at 31.0% vs 39.8%; *P*-value = .007.^[13]^ However, this study was conducted by using VCV mode and did not compare with the PCV mode. The previous systematic review^[4]^ found that there was no significant mortality rate between the VCV and PCV mode with RR of 0.83 (95% CI: 0.67–1.02). Note that the PCV mode was preferable but not significant. This study found that RR was almost significant with 95% CI 1.00 to 1.33 (Fig. 3); PCV mode was preferable than VCV mode.

Several studies also suggested to use low-tidal volume of 6 ml per body weight with a plateau airway pressure of 28 to 30 cm water with an aim to prevent barotrauma.^[4,12–14]^ The previous review did not find different RR of barotrauma by 2 included studies: RR of 1.24 and 95% CI: 0.87–1.77,^[4]^ while this study analyzed by 4 studies and RR was 0.79 (95% CI 0.56–1.12). Both results revealed that it was not significantly different between both ventilations. The previous study^[13]^ using low (6 mL/kg) versus high (12 mL/kg) did not show significant barotrauma (10% vs 11%; *P*-value = .43). These data may imply that tidal volume or pressure of ventilation may not the only factor related with barotrauma.^[15]^ Further studies may be required to explore the causation of barotrauma related to ventilator.

There are some limitations in this study. First, we did not analyzed other factors that may be associated with mortality and/or barotrauma such as comorbid diseases.^[16,17]^ Second, details of VCV or PCV mode were not described or analyzed. Finally, the results of this study were comparable with the previous systematic review.^[4]^ However, this current systematic review had additional details. The study population in this study was both patients with ARDS and COPD, while the previous systematic review included only patients with ARDS. This systematic included both high-income countries and middle-income country (Türkiye), while the previous review included only high-income countries. The VCV mode had a slightly higher mortality outcome than the PCV mode: RR = 1.15; 95% CI: 1.00–1.33 (Fig. 3) in this study but not in the previous study. These differences were due to different statistical method: fixed-effect method in this study vs random-effect method in the previous study. Note that the *I*^2^ was 27%. These results may indicate that PCV mode may have a slightly lower mortality rate than VCV mode in patients with ARDS: 3 studies.

## 5. Conclusion

PCV and VCV had no significant different both barotrauma incidence and mortality rate. PCV mode may have slightly lower mortality and may be preferable ventilator mode in patients with ARDS. Further included studies may be required to confirm the results of this study.

## Author contributions

**Conceptualization:** Sitta Suntrawanichakul, Kittisak Sawanyawisuth, Sittichai Khamsai.

**Data curation:** Sitta Suntrawanichakul, Kittisak Sawanyawisuth, Sittichai Khamsai.

**Searching for potential studies**: Chetta Ngamjarus.

**Formal analysis:** Sitta Suntrawanichakul, Kittisak Sawanyawisuth.

**Writing – original draft:** Sitta Suntrawanichakul, Kittisak Sawanyawisuth, Sittichai Khamsai.

**Writing – review & editing:** Chetta Ngamjarus.

## Supplementary Material



## References

[1] Kempker JA Abril MK Chen Y Kramer MR Waller LA Martin GS. The epidemiology of respiratory failure in the United States 2002–2017: a serial cross-sectional study. Crit Care Explor. 2020;2:e0128. 32695994 10.1097/CCE.0000000000000128 PMC7314331

[2] Hashem MD Nallagangula A Nalamalapu S. Patient outcomes after critical illness: a systematic review of qualitative studies following hospital discharge. Crit Care. 2016;20:345. 27782830 10.1186/s13054-016-1516-x PMC5080744

[3] Hickey SM Giwa AO. Mechanical ventilation. In: StatPearls. StatPearls Publishing; 2024. http://www.ncbi.nlm.nih.gov/books/NBK539742/. Accessed March 30, 2024. 30969564

[4] Chacko B Peter JV Tharyan P John G Jeyaseelan L. Pressure-controlled versus volume-controlled ventilation for acute respiratory failure due to acute lung injury (ALI) or acute respiratory distress syndrome (ARDS). Cochrane Database Syst Rev. 2015;1:CD008807. 25586462 10.1002/14651858.CD008807.pub2 PMC6457606

[5] Mendeley – Reference Management Software. https://www.mendeley.com/. Accessed May 4, 2024.

[6] Higgins JP Thomas J Chandler J, ed, . Cochrane Handbook for Systematic Reviews of Interventions Version 6.5. Cochrane; 2024. cochrane.org/handbook.

[7] RevMan. https://training.cochrane.org/online-learning/core-software/revman. Accessed May 4, 2024.

[8] Meade MO Cook DJ Guyatt GH; Lung Open Ventilation Study Investigators. Ventilation strategy using low tidal volumes, recruitment maneuvers, and high positive end-expiratory pressure for acute lung injury and acute respiratory distress syndrome: a randomized controlled trial. JAMA. 2008;299:637–45. 18270352 10.1001/jama.299.6.637

[9] Esteban A Alía I Gordo F. Prospective randomized trial comparing pressure-controlled ventilation and volume-controlled ventilation in ARDS. For the Spanish Lung Failure Collaborative Group. Chest. 2000;117:1690–6. 10858404 10.1378/chest.117.6.1690

[10] Rappaport SH Shpiner R Yoshihara G Wright J Chang P Abraham E. Randomized, prospective trial of pressure-limited versus volume-controlled ventilation in severe respiratory failure. Crit Care Med. 1994;22:22–32. 8124968 10.1097/00003246-199401000-00009

[11] Ugurlucan M Basaran M Erdim F. Pressure-controlled mechanical ventilation is more advantageous in the follow-up of patients with chronic obstructive pulmonary disease after open heart surgery. Heart Surg Forum. 2014;17:E1–6. 24631983 10.1532/HSF98.2013236

[12] Katira BH. Ventilator-induced lung injury: classic and novel concepts. Respir Care. 2019;64:629–37. 31110032 10.4187/respcare.07055

[13] Brower RG Matthay MA Morris A Schoenfeld D Thompson BT Wheeler A; Acute Respiratory Distress Syndrome Network. Ventilation with lower tidal volumes as compared with traditional tidal volumes for acute lung injury and the acute respiratory distress syndrome. N Engl J Med. 2000;342:1301–8. 10793162 10.1056/NEJM200005043421801

[14] Slutsky AS Ranieri VM. Ventilator-induced lung injury. N Engl J Med. 2013;369:2126–36. 24283226 10.1056/NEJMra1208707

[15] Ioannidis G Lazaridis G Baka S. Barotrauma and pneumothorax. J Thorac Dis. 2015;7(Suppl 1):S38–43. 25774306 10.3978/j.issn.2072-1439.2015.01.31 PMC4332090

[16] Charoentanyarak S Sawunyavisuth B Deepai S Sawanyawisuth K. A point-of-care serum lactate level and mortality in adult sepsis patients: a community hospital setting. J Prim Care Community Health. 2021;12:21501327211000233. 33733925 10.1177/21501327211000233 PMC7983462

[17] Tongdee S Sawunyavisuth B Sukeepaisarnjaroen W Boonsawat W Khamsai S Sawanyawisuth K. Clinical factors predictive of appropriate treatment in COPD: a community hospital setting. Drug Target Insights. 2021;15:21–5. 34803374 10.33393/dti.2021.2291 PMC8600449

