# Supplementary material for: Pressure-controlled ventilation versus volume-controlled ventilation for adult patients with acute respiratory failure: A systematic review and meta-analysis
Source: Medicine (Baltimore). 2025 Aug 22;104(34):e43774. doi: 10.1097/MD.0000000000043774 (PMC12384858; doi:10.1097/MD.0000000000043774)
Supplement: Supplementary file 1 [file medi-104-e43774-s001.docx]

**Appendix**

Appendix 1: Searching method for PubMed (on 14 Jul 2023)

| **Search number** | **Query** |
| --- | --- |
| 1 | ("Respiration, Artificial"[Mesh] OR mechanical ventilation[Title/Abstract]) AND intubat*[Title/Abstract] |
| 2 | (volume control*[Title/Abstract]) OR ventilation[Title/Abstract] OR (VCV[Title/Abstract]) OR (equivalent volume-control*[Title/Abstract]) |
| 3 | (pressure-control*[Title/Abstract]) OR (inverse-ratio[Title/Abstract]) OR (PCV[Title/Abstract]) |
| 4 | ("Pneumothorax"[Mesh]) OR "Barotrauma"[Mesh] |
| 5 | #1 AND #2 AND #3 AND #4 |

Appendix 2: Searching method for Central (on 14 Jul 2023)

| **Search number** | **Query** |
| --- | --- |
| 1 | MeSH descriptor: [Respiration, Artificial] explode all trees |
| 2 | (volume control*):ti,ab,kw OR (ventilation):ti,ab,kw OR (VCV):ti,ab,kw OR (equivalent volume-control*):ti,ab,kw |
| 3 | (pressure-control*):ti,ab,kw OR (inverse-ratio):ti,ab,kw OR (PCV):ti,ab,kw |
| 4 | MeSH descriptor: [Pneumothorax] explode all trees |
| 5 | MeSH descriptor: [Barotrauma] explode all trees |
| 6 | (#1 AND #2 AND #3) AND (#4 OR #5) |

Appendix 3: Searching method for Scopus (on 14 Jul 2023)

TITLE-ABS-KEY ( respiration ) AND ( TITLE-ABS-KEY ( volume AND control* ) OR TITLE-ABS-KEY ( ventilation ) OR TITLE-ABS-KEY ( vcv ) OR TITLE-ABS-KEY ( equivalent AND volume-control* ) ) AND ( TITLE-ABS-KEY ( pressure-control* ) OR TITLE-ABS-KEY ( inverse-ratio ) OR TITLE-ABS-KEY ( pcv ) ) AND ( TITLE-ABS-KEY ( pneumothorax ) OR TITLE-ABS-KEY ( barotrauma ) )4

Appendix 4: Searching method for CINAHL (on 14 Jul 2023)

| **Search number** | **Query** |
| --- | --- |
| S1 | TI respiration OR AB respiration  Expanders - Apply equivalent subjects  Search modes - Find all my search terms |
| S2 | TI volume control ventilation OR AB volume control ventilation OR AB VCV OR AB equivalent  Expanders - Apply equivalent subjects  Search modes - Find all my search terms |
| S3 | TI pressure control ventilation OR AB pressure control ventilation OR AB inverse-ratio OR AB PCV  Expanders - Apply equivalent subjects  Search modes - Find all my search terms |
| S4 | TI Pneumothorax OR AB Pneumothorax OR TI Barotrauma OR AB Barotrauma  Expanders - Apply equivalent subjects  Search modes - Find all my search terms |
| S5 | S1 AND S2 AND S3 AND S4 |
